# Orchestration of B and T cells predicts prolonged survival after cancer immune checkpoint inhibitor therapy

**DOI:** 10.1038/s41698-026-01427-9

**Published:** 2026-04-28

**Authors:** Yu Fujiwara, Shumei Kato, Daisuke Nishizaki, Hirotaka Miyashita, Suzanna Lee, Taylor J. Jensen, Paul DePietro, RJ Seager, Sadakatsu Ikeda, Razelle Kurzrock

**Affiliations:** 1 Department of Medicine, Roswell Park Comprehensive Cancer Center, Buffalo, NY USA; 2 Department of Clinical Oncology, Graduate School of Medical and Dental Sciences, Institute of Science Tokyo, Tokyo, Japan; 3 Division of Hematology-Oncology, Department of Medicine, Moores Cancer Center, University of California San Diego, La Jolla, CA USA; 4 Labcorp, Durham, NC USA; 5 MCW Cancer Center and Genomic Sciences and Precision Medicine Center, Medical College of Wisconsin, Milwaukee, WI USA; 6 WIN Consortium, Paris, France; 7 Department of Oncology, University of Nebraska, Omaha, NE USA

**Keywords:** Biomarkers, Cancer, Computational biology and bioinformatics, Immunology, Oncology

## Abstract

Tertiary lymphoid structures (TLSs) facilitate tumor microenvironment immune interactions. While TLSs and their associated gene signatures correlate with better response to immune checkpoint inhibitors (ICIs), the complex transcriptomic interplay/co-regulation among these factors remains under investigation. We analyzed correlations between survival and transcriptome expression (35 immunoregulatory factors associated with B/T cells and TLSs) in 217 patients with ICI-treated solid tumors. Denser correlations among B/T cell markers, immune checkpoints, and TLS-related molecules was associated with longer survival. High CXCL13 (B-lymphocyte chemoattractant) expression correlated with longer overall survival (hazard ratio [HR] 0.46 [95% CI: 0.27–0.81], *p* = 0.006) in 217 ICI-treated patients but not in 272 ICI-naïve patients (HR 0.85 [0.52–1.40], *p* = 0.519), suggesting its potential predictive/ICI-related, rather than prognostic, value. Overall, patients with highly coordinated T- and B-cell activity, checkpoints, and TLS-related molecules, and higher CXCL13 expression demonstrated prolonged survival after ICIs. Comprehensive multiomics and tumor immunomic profiling may better predict outcome after immunotherapy.

## Introduction

Immunotherapy, particularly immune checkpoint inhibitors (ICIs), has become a standard of care for patients with cancer. ICIs can provide a durable response with prolonged survival, which led to approvals by the U.S. Food and Drug Administration (FDA) for a variety of cancer types^1^. However, most tumors are resistant to ICIs and, thus, it is crucial to identify biomarkers that predict prolonged survival after ICI therapy and to elucidate immune milieus contributing to treatment response versus resistance in the tumor microenvironment (TME).

Over the past decade, several biomarkers, such as programmed death-ligand 1 (PD-L1) (or programmed cell death protein 1 [PD-1]) positivity via immunohistochemistry (IHC), increased tumor-infiltrating lymphocytes (TILs) in the TME, high tumor mutational burden (TMB), and microsatellite instability (MSI) status, have been shown to be associated with prolonged survival following ICIs^2–5^. However, the complexity of the TME and response/resistance to various immunotherapies indicates that additional biomarkers are needed. Additionally, understanding the mechanisms of resistance to ICIs could facilitate the identification of appropriate therapeutic options such as combination treatments involving ICIs and other agents targeting resistance mechanisms^6^.

Immunomodulatory factors, such as immunogenicity of tumor neoantigens, major histocompatibility complex (MHC) class I types, and activated signaling pathways associated with cell proliferation, as well as an immune-suppressive niche characterized by the recruitment of regulatory T cells, M2-like tumor-associated macrophages, and myeloid-derived suppressor cells, have been proposed as mechanisms mediating response and resistance to ICI therapy^7–10^. These elements in the TME dynamically interact with each other, creating complexity and heterogeneity in immune compositions among individual patients. Therefore, a more comprehensive analysis of the network of immunoregulatory factors, which could lead to the identification of more precise biomarkers and signatures for predicting the efficacy of ICIs, is needed to ultimately enable a tailored and personalized therapeutic approach^1,11^.

Tumor-infiltrating effector T cells appear essential for the therapeutic efficacy of ICIs^12^. In fact, therapy involving TILs provides benefit in certain tumors such as malignant melanoma, indicating that the increased cytotoxic T cells in the TME can enhance anti-tumor immunity^13^. However, the presence of tumor-infiltrating T cells alone has not demonstrated a strong correlation with response to ICI therapy; immunohistochemical staining of CD8+ T cells does not appear to be a consistently strong biomarker across tumor types^14–16^. Thus, immune components that recruit these cells, as well as other factors contributing to anti-tumor immunity, such as tumor antigen presentation, have been investigated as potentially more reliable biomarkers^1,12,17^.

The TME that provides a functional cancer immunity cycle, including tumor neoantigen presentation and the migration of TILs, likely ensures anti-tumor immunity. ICIs may be more effective in such an environment if immune suppression is primarily driven by increased expression of co-inhibitory immune checkpoints^18^. More recently, the interaction between antigen-presenting cells, including B cells and dendritic cells, and T cells has been proposed as an essential process, and tertiary lymphoid structures (TLSs) have been posited as an important niche that supports the cancer immunity cycle^19^. Several studies have reported that the presence of TLSs, either morphologically or defined as high expression of TLS gene signatures such as 12CK and Imprint, is associated with better prognosis and longer survival after ICI therapy in patients with tumors^20–25^. However, studies investigating factors that contribute to the formation of TLSs, such as circulating or tumor-intrinsic pivotal chemokines including CXCL13, CCL19, and CCL21, as well as interactions between effector T cells and other antigen-presenting cells at the molecular level and their effect on prognosis and cancer immunotherapy efficacy, remain scarce^20^.

Given that lymphoid structures appear to play a crucial role in anti-tumor immunity, our study aimed to leverage transcriptome expression to elucidate correlations among immunoregulatory factors, including markers for B cells and TLSs, in order to assess their association with ICI therapy outcome.

## Results

### Clinical characteristics

Amongst the 217 patients treated with ICIs, 208 patients with confirmation of disease progression and 180 patients with confirmation of OS were first analyzed. In the PFS group, 82 patients had a PFS of 6 months or more, and 126 patients had a PFS of less than 6 months. In the OS group, 80 patients had an OS of 1 year or more, and 100 patients had an OS of less than 1 year. Patients in the shorter PFS group were younger than those in the longer PFS group. MSI-high status was more frequently observed in the longer PFS and OS groups. Other clinical characteristics were well balanced between the longer and shorter PFS and OS groups (Supplementary Tables 2, 3).

### Transcriptome expression of selected B and T cell immunoregulatory factors shows correlation between higher CXCL13 and better outcome after ICI

mRNA expression of selected immune markers was compared between the longer and shorter post-ICI survival groups. In univariate analysis, high transcriptome expression of CXCL13, CD3, BTLA, CTLA-4, and PD-1 was more frequently observed in the longer PFS group, and high expression of CD80, CD38, CXCL13, IL-10, IDO1, CD137, ICOS, BTLA, CTLA-4, LAG-3, PD-1, TIGIT, and TIM3 was more frequently observed in the longer OS group. In multivariable analysis incorporating factors with a *p* value of less than 0.1 in univariate analysis, none of these factors were associated with longer or shorter survival. In multivariable analysis, incorporating all factors evaluated in univariate analysis, high expression of CXCL13 alone was more frequently observed in the longer PFS and OS groups (PFS analysis: OR = 3.91, 95% confidence interval [CI]: 1.02–15.04, *p* = 0.044; OS analysis: OR = 14.08, 95% CI: 1.80–110.3, *p* = 0.012) (Tables 1, 2).

**Table 1:** B and T cell marker analysis between longer progression-free survival (6 months or more, *N* = 82) and shorter progression-free survival (less than 6 months, *N* = 126) to immune checkpoint inhibitors (*N* = 208)

Category of immune markers	Immune markers		*N* of patients (%)	Longer vs Shorter PFS (%)		OR (95% CI), univariate		Adjusted OR (95% CI), multivariable analysis using factors with *p* < 0.1 in univariate analysis		Adjusted OR (95% CI), multivariable analysis using all factors used in univariate analysis	
			*N* = 208^a^	Longer PFS *N* = 82	Shorter PFS *N* = 126		*p* value^b^		*p* value		*p* value^b^
B cell	CD19	H	29 (13.9)	14 (17.1)	15 (11.9)	1.52 (0.69, 3.35)	0.312			0.85 (0.2, 3.68)	0.829
		I/L	179 (86.1)	68 (82.9)	111 (88.1)						
B cell	CD20	H	31 (14.9)	15 (18.3)	16 (12.7)	1.54 (0.71, 3.31)	0.32			1.00 (0.26, 3.84)	0.997
		I/L	177 (85.1)	67 (81.7)	110 (87.3)						
B cell	CD79A	H	35 (16.8)	16 (19.5)	19 (15.1)	1.37 (0.66, 2.84)	0.45			1.57 (0.37, 6.69)	0.545
		I/L	173 (83.2)	66 (80.5)	107 (84.9)						
B cell	CD80	H	44 (21.2)	17 (20.7)	27 (21.4)	0.96 (0.48, 1.9)	1			0.88 (0.25, 3.09)	0.844
		I/L	164 (78.8)	65 (79.3)	99 (78.6)						
B cell, tertiary lymphoid structure	IL-21	H	20 (9.6)	8 (9.8)	12 (9.5)	1.03 (0.4, 2.63)	1			0.3 (0.06, 1.58)	0.154
		I/L	188 (90.4)	74 (90.2)	114 (90.5)						
B cell, plasma cell	CD27	H	40 (19.2)	19 (23.2)	21 (16.7)	1.51 (0.75, 3.02)	0.282			0.46 (0.09, 2.41)	0.356
		I/L	168 (80.8)	63 (76.8)	105 (83.3)						
Plasma cell	CD38	H	30 (14.4)	15 (18.3)	15 (11.9)	1.66 (0.76, 3.6)	0.228			0.75 (0.19, 2.9)	0.677
		I/L	178 (85.6)	67 (81.7)	111 (88.1)						
Plasma cell	TP63	H	23 (11.1)	10 (12.2)	13 (10.3)	1.21 (0.5, 2.9)	0.659			1.00 (0.32, 3.13)	0.997
		I/L	185 (88.9)	72 (87.8)	113 (89.7)						
Tertiary lymphoid structure	CCL21	H	42 (20.2)	16 (19.5)	26 (20.6)	0.93 (0.46, 1.87)	1			0.57 (0.21, 1.54)	0.264
		I/L	166 (79.8)	66 (80.5)	100 (79.4)						
Tertiary lymphoid structure	CXCL13	H	34 (16.3)	21 (25.6)	13 (10.3)	2.99 (1.4, 6.39)	**0.007**	2.29 (0.85, 6.21)	0.102	3.98 (1.03, 15.34)	**0.045 - CXCL13 was more common with longer PFS**
		I/L	174 (83.7)	61 (74.4)	113 (89.7)						
Regulatory B cell	CD40	H	43 (20.7)	17 (20.7)	26 (20.6)	1.01 (0.51, 2)	1			0.57 (0.21, 1.52)	0.259
		I/L	165 (79.3)	65 (79.3)	100 (79.4)						
Regulatory B cell	GZMB	H	33 (15.9)	14 (17.1)	19 (15.1)	1.16 (0.55, 2.47)	0.702			0.38 (0.1, 1.41)	0.147
		I/L	175 (84.1)	68 (82.9)	107 (84.9)						
Regulatory B cell	IL-10	H	38 (18.3)	17 (20.7)	21 (16.7)	1.31 (0.64, 2.66)	0.468			1.34 (0.41, 4.37)	0.623
		I/L	170 (81.7)	65 (79.3)	105 (83.3)						
Regulatory B cell	TGFB1	H	54 (26.0)	19 (23.2)	35 (27.8)	0.78 (0.41, 1.49)	0.519			0.77 (0.29, 2.01)	0.587
		I/L	154 (74.0)	63 (76.8)	91 (72.2)						
Regulatory B cell	TNF	H	56 (26.9)	19 (23.2)	37 (29.4)	0.73 (0.38, 1.38)	0.342			0.45 (0.19, 1.08)	0.073
		I/L	152 (73.1)	63 (76.8)	89 (70.6)						
T cell	CD3	H	38 (18.3)	20 (24.4)	18 (14.3)	1.94 (0.95, 3.93)	**0.07**	0.71 (0.22, 2.32)	0.567	3.28 (0.58, 18.49)	0.178
		I/L	170 (81.7)	62 (75.6)	108 (85.7)						
T cell	CD4	H	41 (19.7)	16 (19.5)	25 (19.8)	0.98 (0.49, 1.97)	1			0.67 (0.19, 2.35)	0.531
		I/L	167 (80.3)	66 (80.5)	101 (80.2)						
T cell	CD8	H	42 (20.2)	21 (25.6)	21 (16.7)	1.72 (0.87, 3.41)	0.157			0.82 (0.17, 3.93)	0.802
		I/L	167 (80.3)	61 (74.4)	105 (83.3)						
Regulatory T cell	FOXP3	H	54 (26.0)	22 (26.8)	32 (25.4)	1.08 (0.57, 2.03)	0.872			0.78 (0.25, 2.43)	0.664
		I/L	154 (74.0)	60 (73.2)	94 (74.6)						
Immunosuppressive metabolite	IDO1	H	47 (22.6)	24 (29.3)	23 (18.3)	1.85 (0.96, 3.57)	**0.089**	1.33 (0.63, 2.81)	0.462	2.36 (0.88, 6.31)	0.087
		I/L	161 (77.4)	58 (70.7)	103 (81.7)						
Co-stimulatory checkpoint	CD137	H	39 (18.8)	19 (23.2)	20 (15.9)	1.6 (0.79, 3.22)	0.206			1.31 (0.36, 4.7)	0.683
		I/L	169 (81.3)	63 (76.8)	106 (84.1)						
Co-stimulatory checkpoint	CD28	H	38 (18.3)	16 (19.5)	22 (17.5)	1.15 (0.56, 2.34)	0.717			0.31 (0.07, 1.27)	0.103
		I/L	170 (81.7)	66 (80.5)	104 (82.5)						
Co-stimulatory checkpoint	CD40LG	H	39 (18.8)	18 (22.0)	21 (16.7)	1.41 (0.7, 2.84)	0.367			0.93 (0.34, 2.57)	0.893
		I/L	169 (81.3)	64 (78.0)	105 (83.3)						
Co-stimulatory checkpoint	CD70	H	36 (17.3)	16 (19.5)	20 (15.9)	1.28 (0.62, 2.65)	0.575			2.07 (0.8, 5.32)	0.132
		I/L	172 (82.7)	66 (80.5)	106 (84.1)						
Co-stimulatory checkpoint	ICOS	H	28 (13.5)	15 (18.3)	13 (10.3)	1.95 (0.87, 4.34)	0.144			1.52 (0.31, 7.54)	0.607
		I/L	180 (86.5)	67 (81.7)	113 (89.7)						
Co-stimulatory checkpoint	OX40	H	45 (21.6)	18 (22.0)	27 (21.4)	1.03 (0.53, 2.02)	1			0.62 (0.2, 1.89)	0.398
		I/L	163 (78.4)	64 (78.0)	99 (78.6)						
Inhibitory checkpoint	BTLA	H	34 (16.3)	19 (23.2)	15 (11.9)	2.23 (1.06, 4.7)	**0.036**	1.55 (0.49, 4.95)	0.455	1.71 (0.34, 8.66)	0.517
		I/L	174 (83.7)	63 (76.8)	111 (88.1)						
Inhibitory checkpoint	CTLA-4	H	38 (18.3)	21 (25.6)	17 (13.5)	2.21 (1.08, 4.5)	**0.042**	1.2 (0.44, 3.25)	0.718	2.14 (0.49, 9.26)	0.31
		I/L	170 (81.7)	61 (74.4)	109 (86.5)						
Inhibitory checkpoint	LAG-3	H	51 (24.5)	24 (29.3)	27 (21.4)	1.52 (0.8, 2.87)	0.248			1.05 (0.41, 2.68)	0.926
		I/L	157 (75.5)	58 (70.7)	99 (78.6)						
Inhibitory checkpoint	PD-1	H	43 (20.7)	23 (28.0)	20 (15.9)	2.07 (1.05, 4.07)	**0.037**	1.14 (0.42, 3.08)	0.799	1.93 (0.58, 6.49)	0.286
		I/L	165 (79.3)	59 (72.0)	106 (84.1)						
Inhibitory checkpoint	PD-L1	H	35 (16.8)	16 (19.5)	19 (15.1)	1.37 (0.66, 2.84)	0.45			1.24 (0.35, 4.41)	0.742
		I/L	173 (83.2)	66 (80.5)	107 (84.9)						
Inhibitory checkpoint	PD-L2	H	49 (23.6)	23 (28.0)	26 (20.6)	1.5 (0.79, 2.86)	0.244			1.62 (0.5, 5.23)	0.417
		I/L	159 (76.4)	59 (72.0)	100 (79.4)						
Inhibitory checkpoint	TIGIT	H	37 (17.8)	18 (22.0)	19 (15.1)	1.58 (0.77, 3.24)	0.265			0.38 (0.05, 2.66)	0.327
		I/L	171 (82.2)	64 (78.0)	107 (84.9)						
Inhibitory checkpoint	TIM3	H	43 (20.7)	20 (24.4)	23 (18.3)	1.44 (0.73, 2.84)	0.298			2.15 (0.56, 8.17)	0.263
		I/L	165 (79.3)	62 (75.6)	103 (81.7)						
Inhibitory checkpoint	VISTA	H	57 (27.4)	25 (30.5)	32 (25.4)	1.29 (0.69, 2.39)	0.431			1.54 (0.62, 3.8)	0.35
		I/L	151 (72.6)	57 (69.5)	94 (74.6)						

*H* high, *I/L* intermediate/low, *OR* odds ratio, *PFS* progression-free survival.

^a^9 out of 217 patients who lost to follow-up within 6 months without confirmation of progression of disease after initiation of immune checkpoint inhibitors were excluded. All patients censored before 6 months were excluded from this analysis. ^b^*p* values of 0.1 or less for the univariate analysis and *p* values of 0.05 or less for the multivariable analyses are shown in bold.

**Table 2:** B and T cell marker analysis between longer overall survival (1 year or more, *N* = 80) and shorter overall survival (less than 1 year, *N* = 100) to immune checkpoint inhibitors (*N* = 180)

Category of immune markers	Immune markers		*N* of patients (%)	Longer vs Shorter OS (%)		OR (95% CI), univariate		Adjusted OR (95% CI), multivariable analysis using factors with *p* < 0.1 in univariate analysis		Adjusted OR (95% CI), multivariable analysis using all factors used in univariate analysis	
			*N* = 180^a^	Longer OS *N* = 80	Shorter OS *N* = 100		*p* value^b^		*p* value		*p* value^b^
B cell	CD19	H	26 (14.4)	16 (61.5)	10 (38.5)	1.33 (0.57, 3.12)	0.531			0.3 (0.05, 1.99)	0.214
		I/L	154 (85.6)	84 (54.5)	70 (45.5)						
B cell	CD20	H	27 (15.0)	17 (63.0)	10 (37.0)	1.43 (0.62, 3.33)	0.529			1.75 (0.29, 10.55)	0.543
		I/L	153 (85.0)	83 (54.2)	70 (45.8)						
B cell	CD79A	H	32 (17.8)	19 (59.4)	13 (40.6)	1.21 (0.56, 2.63)	0.697			2.01 (0.32, 12.75)	0.457
		I/L	148 (82.2)	81 (54.7)	67 (45.3)						
B cell	CD80	H	33 (18.3)	25 (75.8)	8 (24.2)	3.00 (1.27, 7.08)	**0.011**	1.77 (0.61, 5.08)	0.291	3.32 (0.68, 16.27)	0.139
		I/L	147 (81.7)	75 (51.0)	72 (49.0)						
B cell, tertiary lymphoid structure	IL-21	H	16 (8.9)	12 (75.0)	4 (25.0)	2.59 (0.80, 8.37)	0.12			0.61 (0.09, 4.08)	0.612
		I/L	164 (91.1)	88 (53.7)	76 (46.3)						
B cell, plasma cell	CD27	H	35 (19.4)	22 (62.9)	13 (37.1)	1.45 (0.68, 3.11)	0.351			0.46 (0.06, 3.47)	0.455
		I/L	145 (80.6)	78 (53.8)	67 (46.2)						
Plasma cell	CD38	H	26 (14.4)	20 (76.9)	6 (23.1)	3.08 (1.17, 8.10)	**0.019**	1.04 (0.3, 3.6)	0.95	0.74 (0.16, 3.43)	0.705
		I/L	154 (85.6)	80 (51.9)	74 (48.1)						
Plasma cell	TP63	H	18 (10.0)	10 (55.6)	8 (44.4)	1.00 (0.38, 2.66)	1			0.31 (0.06, 1.45)	0.135
		I/L	162 (90.0)	90 (55.6)	72 (44.4)						
Tertiary lymphoid structure	CCL21	H	40 (22.2)	21 (52.5)	19 (47.5)	0.85 (0.42, 1.73)	0.72			0.69 (0.22, 2.11)	0.514
		I/L	140 (77.8)	79 (56.4)	61 (43.6)						
Tertiary lymphoid structure	CXCL13	H	27 (15.0)	22 (81.5)	5 (18.5)	4.23 (1.52, 11.75)	**0.003**	2.68 (0.72, 9.99)	0.141	14.08 (1.8, 110.34)	**0.012 - CXCL13 was more common with longer OS**
		I/L	153 (85.0)	78 (51.0)	75 (49.0)						
Regulatory B cell	CD40	H	34 (18.9)	22 (64.7)	12 (35.3)	1.60 (0.74, 3.47)	0.256			0.79 (0.23, 2.72)	0.704
		I/L	146 (81.1)	78 (53.4)	68 (46.6)						
Regulatory B cell	GZMB	H	27 (15.0)	17 (63.0)	10 (37.0)	1.43 (0.62, 3.33)	0.529			0.23 (0.05, 1.14)	0.072
		I/L	153 (85.0)	83 (54.2)	70 (45.8)						
Regulatory B cell	IL-10	H	31 (17.2)	23 (74.2)	8 (25.8)	2.69 (1.13, 6.39)	**0.028**	1.53 (0.49, 4.74)	0.461	3.34 (0.7, 15.9)	0.13
		I/L	149 (82.8)	77 (51.7)	72 (48.3)						
Regulatory B cell	TGFB1	H	42 (23.3)	22 (52.4)	20 (47.6)	0.85 (0.42, 1.69)	0.723			0.42 (0.11, 1.56)	0.196
		I/L	138 (76.7)	78 (56.5)	60 (43.5)						
Regulatory B cell	TNF	H	45 (25.0)	29 (64.4)	16 (35.6)	1.63 (0.81, 3.28)	0.225			0.93 (0.32, 2.75)	0.897
		I/L	135 (75.0)	71 (52.6)	64 (47.4)						
T cell	CD3	H	32 (17.8)	22 (68.8)	10 (31.2)	1.97 (0.87, 4.46)	0.118			0.27 (0.03, 2.67)	0.264
		I/L	148 (82.2)	78 (52.7)	70 (47.3)						
T cell	CD4	H	36 (20.0)	23 (63.9)	13 (36.1)	1.54 (0.72, 3.27)	0.349			0.43 (0.08, 2.25)	0.315
		I/L	144 (80.0)	77 (53.5)	67 (46.5)						
T cell	CD8	H	37 (20.6)	24 (64.9)	13 (35.1)	1.63 (0.77, 3.45)	0.265			0.41 (0.07, 2.22)	0.298
		I/L	143 (79.4)	76 (53.1)	67 (46.9)						
Regulatory T cell	FOXP3	H	45 (25.0)	28 (62.2)	17 (37.8)	1.44 (0.72, 2.88)	0.387			0.52 (0.13, 1.97)	0.334
		I/L	135 (75.0)	72 (53.3)	63 (46.7)						
Immunosuppressive metabolite	IDO1	H	33 (18.3)	26 (78.8)	7 (21.2)	2.32 (1.07, 5.03)	**0.042**	1.34 (0.52, 3.47)	0.544	1.82 (0.56, 5.88)	0.32
		I/L	147 (81.7)	74 (50.3)	73 (49.7)						
Co-stimulatory checkpoint	CD137	H	34 (18.9)	22 (64.7)	12 (35.3)	3.66 (1.50, 8.97)	**0.003**	1.92 (0.57, 6.42)	0.292	3.63 (0.73, 18.12)	0.116
		I/L	146 (81.1)	78 (53.4)	68 (46.6)						
Co-stimulatory checkpoint	CD28	H	36 (20.0)	24 (66.7)	12 (33.3)	1.60 (0.74, 3.47)	0.256			0.28 (0.06, 1.42)	0.125
		I/L	144 (80.0)	76 (52.8)	68 (47.2)						
Co-stimulatory checkpoint	CD40LG	H	30 (16.7)	19 (63.3)	11 (36.7)	1.79 (0.83, 3.85)	0.189			1.79 (0.52, 6.21)	0.356
		I/L	150 (83.3)	81 (54.0)	69 (46.0)						
Co-stimulatory checkpoint	CD70	H	23 (12.8)	19 (82.6)	4 (17.4)	1.47 (0.66, 3.30)	0.423			1.52 (0.47, 4.94)	0.488
		I/L	157 (87.2)	81 (51.6)	76 (48.4)						
Co-stimulatory checkpoint	ICOS	H	36 (20.0)	21 (58.3)	15 (41.7)	4.46 (1.45, 13.70)	**0.006**	1.83 (0.34, 9.77)	0.477	3.32 (0.36, 30.94)	0.293
		I/L	144 (80.0)	79 (54.9)	65 (45.1)						
Co-stimulatory checkpoint	OX40	H	31 (17.2)	23 (74.2)	8 (25.8)	1.15 (0.55, 2.41)	0.852			0.95 (0.24, 3.74)	0.941
		I/L	149 (82.8)	77 (51.7)	72 (48.3)						
Inhibitory checkpoint	BTLA	H	34 (18.9)	26 (76.5)	8 (23.5)	2.69 (1.13, 6.39)	**0.028**	1.18 (0.31, 4.42)	0.81	1.76 (0.31, 9.92)	0.52
		I/L	146 (81.1)	74 (50.7)	72 (49.3)						
Inhibitory checkpoint	CTLA-4	H	45 (25.0)	32 (71.1)	13 (28.9)	3.16 (1.34, 7.44)	**0.007**	1.29 (0.38, 4.45)	0.682	0.89 (0.13, 6.04)	0.902
		I/L	135 (75.0)	68 (50.4)	67 (49.6)						
Inhibitory checkpoint	LAG-3	H	35 (19.4)	25 (71.4)	10 (28.6)	2.43 (1.17, 5.02)	**0.016**	1.48 (0.6, 3.69)	0.395	1.49 (0.5, 4.45)	0.47
		I/L	145 (80.6)	75 (51.7)	70 (48.3)						
Inhibitory checkpoint	PD-1	H	25 (13.9)	18 (72.0)	7 (28.0)	2.33 (1.05, 5.21)	**0.039**	0.9 (0.29, 2.83)	0.863	1.65 (0.43, 6.34)	0.465
		I/L	155 (86.1)	82 (52.9)	73 (47.1)						
Inhibitory checkpoint	PD-L1	H	39 (21.7)	27 (69.2)	12 (30.8)	2.29 (0.90, 5.79)	**0.086**	0.89 (0.23, 3.47)	0.868	0.9 (0.18, 4.59)	0.896
		I/L	141 (78.3)	73 (51.8)	68 (48.2)						
Inhibitory checkpoint	PD-L2	H	31 (17.2)	23 (74.2)	8 (25.8)	2.10 (0.98, 4.46)	**0.068**	0.75 (0.25, 2.22)	0.598	0.91 (0.23, 3.57)	0.892
		I/L	149 (82.8)	77 (51.7)	72 (48.3)						
Inhibitory checkpoint	TIGIT	H	39 (21.7)	29 (74.4)	10 (25.6)	2.69 (1.13, 6.39)	**0.028**	0.42 (0.09, 2.04)	0.282	2.12 (0.21, 21.19)	0.521
		I/L	141 (78.3)	71 (50.4)	70 (49.6)						
Inhibitory checkpoint	TIM3	H	54 (30.0)	33 (61.1)	21 (38.9)	2.86 (1.30, 6.31)	**0.01**	1.29 (0.39, 4.23)	0.675	7.91 (0.98, 63.68)	0.052
		I/L	126 (70.0)	67 (53.2)	59 (46.8)						
Inhibitory checkpoint	VISTA	H	26 (14.4)	16 (61.5)	10 (38.5)	1.38 (0.72, 2.65)	0.413			0.85 (0.28, 2.59)	0.769
		I/L	154 (85.6)	84 (54.5)	70 (45.5)						

*H* high, *I/L* intermediate/low, *OR* odds ratio, *OS* overall survival.

^a^37 out of 217 patients who lost to follow-up within 1 year without confirmation of death after initiation of immune checkpoint inhibitors were excluded. All patients censored before 1 year were excluded from this analysis. ^b^*p* values of 0.1 or less for the univariate analysis and *p* values of 0.05 or less for the multivariable analyses are shown in bold.

### Cluster heatmaps show strong correlations between immune checkpoints, tertiary lymphoid structure (TLS)-related molecules and T cell markers in patients with longer survival post-ICI

Cluster heatmaps illustrated a cluster with tumors homogeneously expressing B and T cell markers in the longer PFS and OS groups, while heterogeneous or low expression of these markers was seen in the shorter PFS and OS groups (Supplementary Fig. 1a–e). Correlation analyses were conducted to further investigate the interactions among factors relevant to B and T cells in the TME within the longer and shorter survival groups. These analyses revealed strong correlations among immune checkpoints (CTLA-4, ICOS, BTLA, TIGIT, and PD-1), factors associated with TLSs (CXCL13 and IL-21), and T cell markers (CD3 and CD8) in the longer survival group. In contrast, correlations were observed among some immune checkpoint markers (BTLA, CTLA-4, ICOS, CD40LG, TIGIT, and CD28), but not among factors related to T cell infiltration or TLSs in the shorter survival group (Supplementary Fig. 2a–e).

**Fig. 1:**
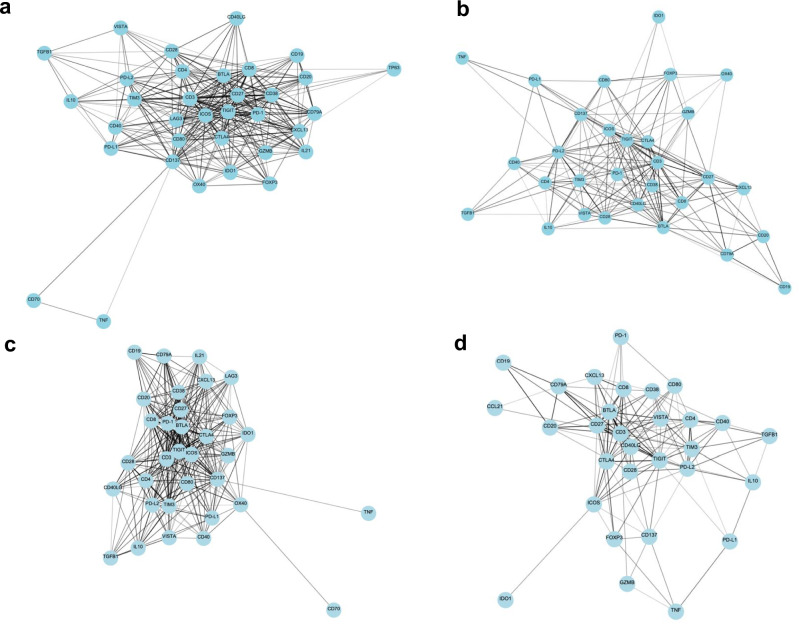
Network diagrams of correlation among B and T cell markers. **a** Patients with progression-free survival of 6 months or more. **b** Patients with progression-free survival of less than 6 months. **c** Patients with overall survival of 1 year or more. **d** Patients with overall survival of less than 1 year. Each line connects a pair of B or T cell markers with a correlation coefficient of 0.5 or higher. Of the total of 595 edges, there are 297 pairs with high correlation in patients with progression-free survival of 6 months or longer (a: *n* = 34 nodes), compared to 172 pairs in patients with progression-free survival of less than 6 months (b: *n* = 30 nodes) (permutation test *p* = 0.085). There are 294 pairs with high correlation in patients with overall survival of 1 year or longer (c: *n* = 33 nodes), compared to 141 pairs in patients with overall survival of less than 1 year (d: *n* = 30 nodes) (*p* = 0.041). Any patients censored before 6 months in the progression-free survival analysis group (**a**, **b**) and those censored before 1 year in the overall survival analysis group (**c**, **d**) were excluded from the analysis.

Network diagrams illustrating moderate or stronger correlations (correlation coefficient > 0.5) demonstrated a denser network of B and T cell factors in the longer OS group than in the shorter OS group (PFS: 0.499 [297/595] vs. 0.289 [172/595], *p* = 0.085; OS: 0.494 [294/595] vs. 0.237 [141/595], *p* = 0.041), suggesting an association between greater interactive expression of these markers and longer OS following ICI therapy (Fig. 1a–d).

### Cancer type and genomic landscape based on CXCL13 mRNA expression

**Fig. 2:**
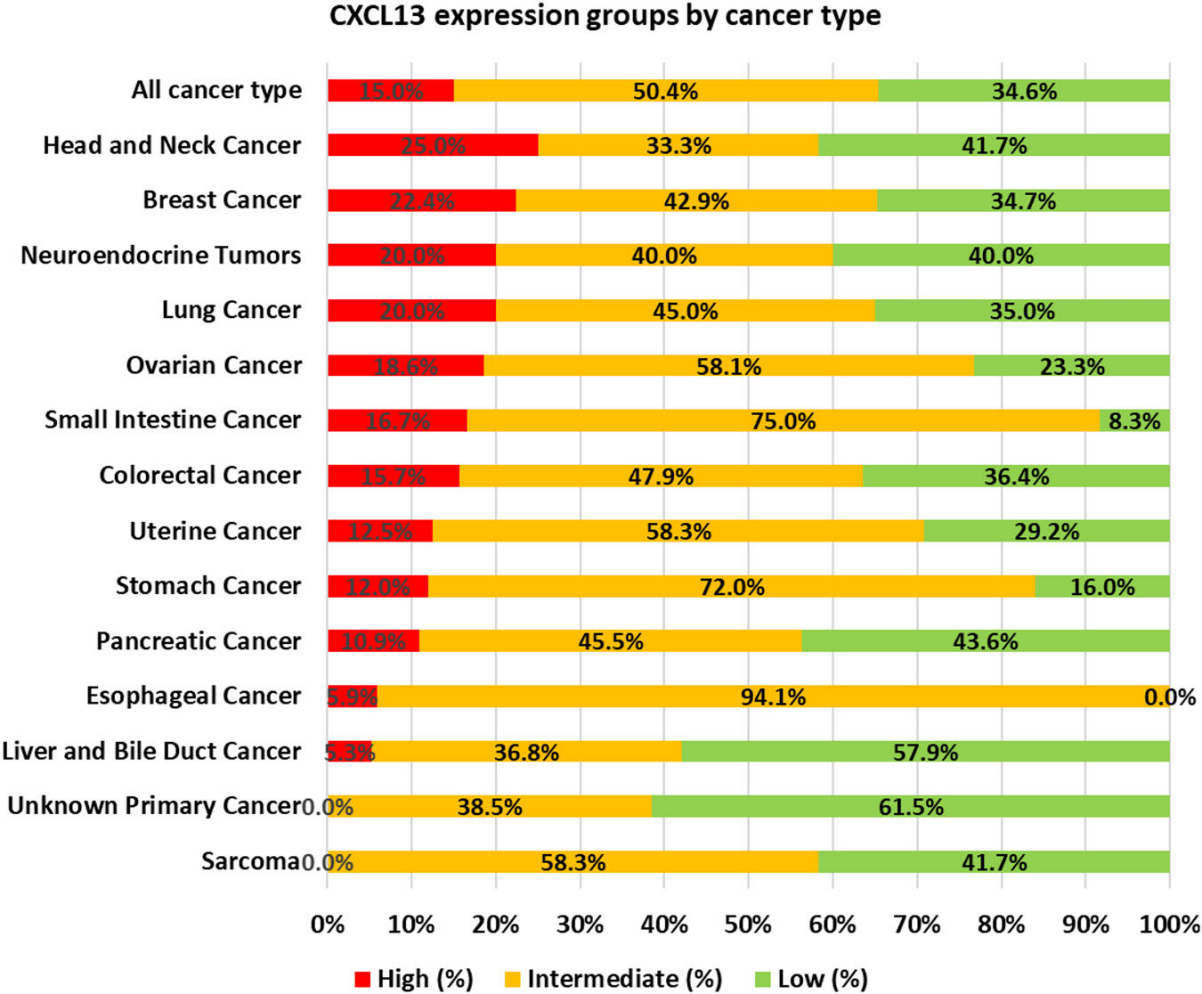
CXCL13 expression groups (high, intermediate, and low) by cancer type (*N* = 514). The percentages on the bar graph indicate the percentage of patients with high (≥75th percentile RNA rank), intermediate (25th to 74th percentile RNA rank), or low (<25th percentile RNA rank) RNA expression. For instance, 15.0% of patients across all cancer types (*N* = 514 patients) had high CXCL13 RNA expression.

As high CXCL13 transcriptome expression was more frequently observed in patients with longer PFS and OS, we further leveraged clinical and genomic features based on CXCL13 expression levels, classifying patients into high (≥75th percentile RNA expression rank), intermediate (25th–74th percentile rank), or low (<25th percentile rank) groups. Of the 514 patients in the entire cohort, 15.0%, 50.4%, and 34.6% had high, intermediate, and low CXCL13 mRNA expression, respectively. High CXCL13 expression was most commonly seen in head and neck cancer (25.0%), followed by breast cancer (22.4%), lung cancer (20.0%), and neuroendocrine tumors (20.0%) (Fig. 2).

**Fig. 3:**
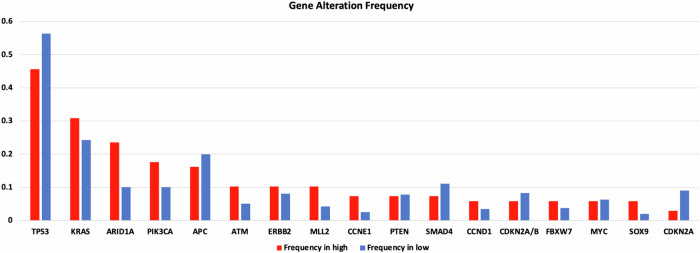
Frequency of gene alterations between high and intermediate/low CXCL13 expression groups. In total, 68 patients in the high CXCL13 expression group and 396 patients in the intermediate/low CXCL13 expression group underwent next-generation sequencing testing. Genes with an alteration frequency of 5% or more in at least one group were selected and compared between these two groups. A Bonferroni adjustment was applied for multiple comparisons, and a *p* value for significance was set at 0.05/17 (=0.00294118). The frequency of gene alterations in each gene was not significantly different between the two CXCL13 expression groups. The *ARID1A* alteration showed a trend toward higher frequency in the high CXCL13 expression group, approaching significance with an adjusted *p* value of 0.067.

Information on genomic alterations was available for 464 patients (high CXCL13 expression: *n* = 68; intermediate/low CXCL13 expression: *n* = 396), and genes with an alteration frequency of 5% or more in at least one group were compared between these two groups. The frequency of gene alterations in selected genes was not significantly different between the two CXCL13 expression groups (Fig. 3). *ARID1A* alterations tended to be more commonly observed in the high CXCL13 group (adjusted *p* = 0.067).

### High CXCL13 transcript expression correlates with longer PFS and OS in ICI-treated patients but does not correlate with outcome in ICI-naïve patients

**Fig. 4:**
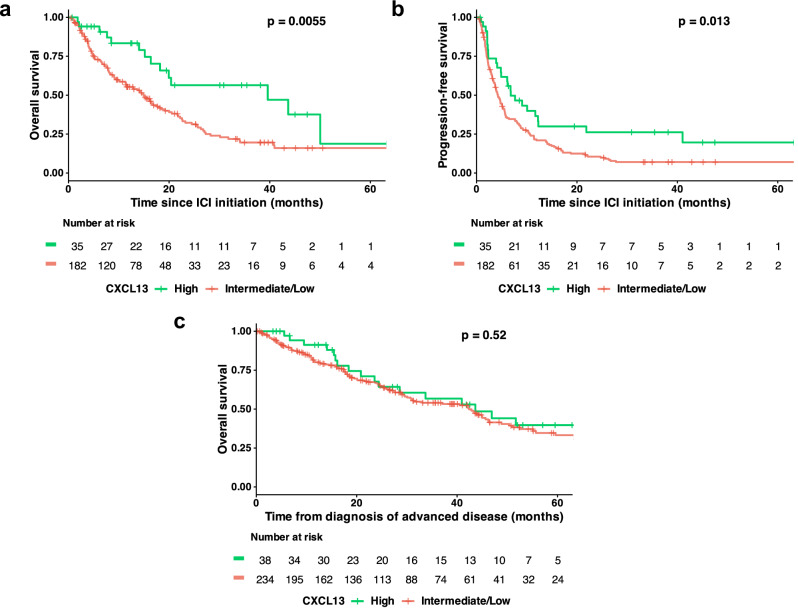
Survival curves based on CXCL13 expression status. Kaplan-Meier curves of **a** overall survival (HR = 0.46, 95% CI 0.27–0.81, *p* = 0.006) and **b** progression-free survival (HR = 0.59, 95% CI 0.38–0.90, *p* = 0.013) in patients treated with immune checkpoint inhibitors (*N* = 217) based on CXCL13 expression. This figure shows that patients with high CXCL13 RNA expression (≥75th percentile rank) had significantly longer OS and PFS from the start time of immunotherapy compared to patients with low to intermediate CXCL13 RNA expression in their tumors. **c** This panel shows Kaplan-Meier curves of overall survival in 272 patients who were not treated with immune checkpoint inhibitors based on CXCL13 expression. This figure shows that there was no significant difference in overall survival from the time of diagnosis of advanced/metastatic disease in patients whose tumors had high (≥75th percentile rank) versus low to intermediate RNA expression (HR = 0.85, 95% CI 0.52–1.40, *p* = 0.519).

In total, 217 patients received ICIs; high CXCL13 mRNA expression was observed in 35 patients, and intermediate/low expression in 182 patients. In these ICI-treated patients, high CXCL13 mRNA expression was associated with longer OS (hazard ratio [HR] = 0.46, 95% CI: 0.27–0.81, *p* = 0.006) and longer PFS (HR = 0.59, 95% CI: 0.38–0.90, *p* = 0.013) compared to intermediate/low CXCL13 mRNA expression (from time of initiation of ICI) (Fig. 4a, b). To further examine the prognostic value of CXCL13 expression, OS was compared between high and intermediate/low CXCL13 expression groups in 272 patients with advanced cancer not treated with ICIs, and no significant difference in OS was observed between these two groups (HR = 0.85, 95% CI: 0.52–1.40, *p* = 0.519) (Fig. 4c). Together, these findings suggest that high CXCL13 expression may serve as a predictive biomarker for ICI therapy but is not a prognostic factor in immunotherapy-naïve patients.

## Discussion

Immunoregulation is based on complex interactions between antigen-presenting cells, including B cells, dendritic cells, and T cells. TLSs are vital to the cancer immunity cycle from tumor neoantigen presentation to the education and maturation of the migrated T cells in the TME^19^. Importantly, CXCL13 is a chemokine that facilitates the organization and formation of TLSs^19,20^. In this study, high CXCL13 transcriptome expression emerged as an important factor, which was more frequently observed in patients with longer survival following ICI therapy compared to those with shorter survival. Correlation analyses also revealed a denser network of immune checkpoints, factors associated with TLSs, and effector T cells in the group of patients with longer survival versus those with shorter survival after ICI, suggesting that close interactions among these immunoregulatory factors enhance the efficacy of ICIs. Our findings align with the established role of TLS-associated gene signatures as predictive biomarkers for ICI therapy. These findings support the concept that the expression of immune checkpoints, an inflamed TME, and TLS-inducing factors are important to outcomes in patients with solid tumors treated with ICIs.

TLSs are specialized structures that provide the environment for immune system training, and studies have reported a positive correlation between their presence and the efficacy of ICIs in solid tumors^21,26,27^. Several immunoregulatory factors, including CXCL13, CCL19, CCL21, and IL-21, recruit antigen-presenting cells, helper and cytotoxic T cells, as well as fibroblasts and follicular dendritic cells to facilitate the formation and maturation of TLSs in the TME^20,28–30^. As such, TLSs have been proposed as an essential niche for ensuring the efficacy of ICIs. Indeed, pathological evaluation of TLS presence has revealed an association with better responses and longer survival in patients receiving ICIs^21^. However, the effect of interactions among these TLS-inducing chemokines and cytokines, and their transcriptional expression levels, on ICI therapy outcomes remains largely unexplored.

Given these observations, TLSs may facilitate local tumor-infiltrating lymphocyte responses by producing cytokines and boosting memory T cell responses to tumor antigens. This process provides an environment where plasma cells secreting tumor-specific antibodies can persist^31^. Therefore, our work interrogated the expression of key B cell markers in addition to TLS-inducing factors and major T cell markers. In our study, we first showed that patients with longer survival after ICI therapy had denser networks of TLS-inducing chemokines and cytokines, co-inhibitory immune checkpoints, as well as pan-B and T cell markers compared to those with shorter survival. These observations support the idea that niches such as TLSs, facilitating interactions among these cells, play an important role in ensuring the efficacy of ICIs. Additional analyses in our study further revealed that patients with longer PFS and OS were more likely to have high mRNA expression of CXCL13. High CXCL13 expression was not associated with longer or shorter survival in patients not treated with ICIs. These findings suggest that, among TLS-inducing immunoregulatory factors, CXCL13 plays a distinct and pivotal role associated with enhanced benefit from ICI therapy.

We further dissected the frequency of gene alterations between high and intermediate/low CXCL13 expression groups to identify potential drivers of CXCL13 mRNA expression. Although our analysis did not reveal statistically significant differences in gene alteration frequency (23.5% vs. 10.1%, adjusted *p* = 0.067), *ARID1A* alterations showed a trend toward higher frequency in the high CXCL13 expression group. This finding aligns with a previous translational study where baseline *ARID1A* mutation and high CXCL13 expression were both associated with improved OS, and the combination of both biomarkers predicted more improved OS compared to either single biomarker alone in two clinical trial cohorts of metastatic urothelial carcinoma treated with ICIs^32^. It also aligns with studies showing that *ARID1A* is a chromatin remodeling gene which, when mutated, is associated with better response to ICIs^33–35^. Since our data demonstrated a trend but no significant differences in *ARID1A* alteration frequency, and literature on how *ARID1A* alteration affects TLS formation remains scarce, further research investigating the mutational landscape of tumors harboring TLSs is needed.

Several limitations should be noted in regard to our study. First, we evaluated curated bulk RNA expression data, which is not specific to a certain cell population in the TME, and our study was not able to estimate specific immune cell populations or identify the distinct roles of immune cells in the TME. Single-cell RNA sequencing or spatial transcriptomics would clarify the dynamics in the TME, as would multiplex immunofluorescence of TLSs and relevant immunoregulatory markers. However, the RNA expression investigated in our work likely reflects the overall composition of the TME. Thus, these specimens serve as meaningful biological materials for leveraging immunoregulatory factors associated with the efficacy of ICIs. A second limitation is that the initial cutoffs for creating subgroups necessitated excluding censored patients in order to maintain accuracy of subgrouping; such an exclusion, however, can introduce selection bias and potentially alter survival estimates. Even so, to mitigate this, once these subgroups were created, subsequent outcome analyses included all patients. Third, some patients with longer survival after ICIs did not exhibit expression patterns associated with prolonged survival; further research needs to address the heterogeneous responses and transcriptomic patterns observed across patients. Additionally, the findings in this study are based on tumor-intrinsic bulk RNA expression without validation via other analytic methods such as proteomics, or the measurement of circulating chemokines which enables convenient and longitudinal assessment of antitumor immunity. This limits our ability to draw definitive conclusions and hinders the translation of transcriptomic insights into clinical practice. Future research should measure circulating chemokines and correlate these with tumor-derived transcriptomics to assess their clinical utility. Notably, CXCL13 correlated with benefit in the ICI-treated patients but not in the ICI-naïve patients; therefore, our data are directionally supportive of a predictive but not prognostic role for CXCL13. However, prospective studies would be needed to confirm this finding. Finally, our study was performed in a tumor-agnostic manner and the impact of cancer type on the TME as well as tumor-type heterogeneity were not fully addressed due to small subgroup sizes, though the results might point to generalizability across cancers.

In conclusion, our work highlights an interactive network and harmonization between T and B cells, the presence of a drug target (i.e., a checkpoint), and an inflamed microenvironment, as key factors associated with the efficacy of ICI therapy. Strategies that modulate the TME into one with a more mature lymphoid structure that supports cancer-associated antigen presentation, the education and migration of T cells, and crosstalk between antigen-presenting cells and effector T cells, might overcome resistance to ICI therapy. CXCL13, a chemokine that facilitates the organization and formation of TLSs, emerged as possibly of particular importance and was more frequently expressed in patients with longer survival following ICI therapy. CXCL13 could be integrated into composite biomarkers along with PD-L1, TMB, and MSI to improve biomarker-based patient selection for ICIs, though prospective validation, including for TLS-related proteins and circulating chemokines as well as for CXCL13 is needed to confirm this and assess real-world applicability. Therapies that modulate the TME to promote immune cell interaction and TLS formation, when evaluated with correlative multiomics analysis, would provide further insights into the potential of these therapeutic interventions. A comprehensive analysis of tumor specimens, rather than relying on a single biomarker such as PD-L1 expression, which has traditionally been used across tumor types, may better predict response to ICI therapy. Such an approach will lead to more personalized treatment approaches in the era of cancer immunotherapy.

## Methods

### Patient population

Patients enrolled in the UCSD-PREDICT study (NCT02478931) with diagnoses of advanced solid tumors were included in our analysis. Of the 514 patients registered in the study, 217 patients with advanced disease received ICIs. To elucidate immunoregulatory factors associated with outcome, we first identified 208 and 180 patients with available progression-free survival (PFS) and overall survival (OS) information, respectively, and divided them into longer-survival (≥6 months for PFS, ≥1 year for OS) and shorter-survival (<6 months for PFS, <1 year for OS) groups. PFS and OS start dates were from the initiation of ICIs; only patients who did not have early censoring (<6 months for PFS and <1 year for OS) were included. Patient clinical characteristics were summarized and compared between the longer and shorter survival groups. The study (NCT02478931) was conducted in accordance with the Declaration of Helsinki, and the study protocol was approved by the Institutional Ethics Board of the University of California, San Diego (UCSD reference number: 130794). All participants provided written informed consent prior to their inclusion in the study.

### Tissue collection, sequencing, and mRNA expression percentile

The tumor specimens of all patients enrolled in the study underwent RNA transcriptome sequencing using a clinically validated gene expression panel. The sequencing method was previously described in detail^17,36–38^. The RNA expression of immunoregulatory factors was calculated, and the transcript abundance of these molecules was normalized and compared to an internal reference population comprising 735 tumors spanning 35 histologies. Rank values for these factors were then generated on a scale from 0 to 100 and categorized as low (rank: 0–24), intermediate (25–74), and high (75–100). Overall, we chose 35 factors associated with response to and resistance to ICIs, including co-inhibitory immune checkpoints, co-stimulatory immune checkpoints, TLSs, B cells, plasma cells, T cells, and immunosuppressive metabolites, to capture the dynamic interactions among these immunoregulatory components (Supplementary Table 1).

### Comparison of mRNA expression between patients with longer and shorter survival

The transcriptomic expression of the selected immunoregulatory markers defined above was compared between patients with longer and shorter PFS and OS. Odds ratios (ORs) for longer survival were calculated based on high vs intermediate/low mRNA expression of each immunoregulatory factor, and Fisher’s exact test was performed for univariate analysis. Subsequently, we performed multivariable analyses using factors with a *p* value of less than 0.1 in univariable analysis, and secondarily using all factors evaluated in univariate analysis, to calculate adjusted ORs with a logistic regression model. Transcriptomic expression of these factors was also visualized by generating heatmaps using the k-means clustering to create four clusters among 514 patients and the ICI-treated population with available PFS and OS information (*n* = 82, PFS ≥ 6 months; *n* = 126, PFS < 6 months; *n* = 80, OS ≥ 1 year; *n* = 100, OS < 1 year).

### Correlation analysis of immunoregulatory markers

Next, we performed a correlation analysis of B and T cell immunoregulatory markers in all 514 patients, including 208 with PFS information (*n* = 82, PFS ≥ 6 months; *n* = 126, PFS < 6 months) and 180 patients with available OS information (*n* = 80, OS ≥ 1 year; *n* = 100, OS < 1 year), to elucidate interactive patterns among these immune factors. A hierarchical clustering method was used to illustrate the correlation matrix, visualizing the landscape of correlations among these factors. Then, we created a network diagram of correlations among B and T cell immunoregulatory factors in patients with longer and shorter survival to evaluate correlative density and patterns. Spearman’s correlation coefficients of 0.5 or higher were chosen to ensure that the network diagram focuses on solid and meaningful relationships between markers and also to avoid an overcrowded diagram resulting from edges with weaker correlations. These relationships were drawn as edges connecting two factors showing strong correlation, and the total number of these edges between the longer-survival and shorter-survival groups was compared using a non-parametric permutation test with 10,000 iterations. In these correlation analyses, any patients who were censored before 6 months were excluded from the PFS group, and those censored before 1 year were excluded from the OS group due to a lack of accurate survival information in these populations.

### Gene alteration frequency between patients with high and low CXCL13 expression

We specifically focused on CXCL13, as it is one of the known chemokines that induce TLSs, which may provide a niche for B and T cell interaction in the lymphoid-like structures around tumor cells^39^. The frequency of gene alterations between the high and non-high CXCL13 expression groups was first compared to capture the genetic makeup according to CXCL13 expression in solid tumors. Overall, 464 out of 514 patients who underwent tissue-based comprehensive genomic profiling using commercially available next-generation sequencing (NGS) platforms (Foundation Medicine, Tempus, and OmniSeq) were evaluable for this analysis. Genes with an alteration frequency of 5% or more in at least one CXCL13 expression group were chosen, and the frequency of these gene alterations was compared using Fisher’s exact test, with a correction using the Bonferroni method (significance threshold: *p* = 0.05/17 = 0.00294118). Additionally, the rate of high, intermediate, and low CXCL13 RNA expression in each cancer type (with ≥10 patients among the 514 included) was calculated and illustrated in a bar graph to capture the histological pattern of CXCL13 RNA expression.

### Survival analysis

Survival outcomes were evaluated based on prespecified clinical and immunoregulatory factors, including age, sex (male versus female), high vs intermediate and low expression of CXCL13, PD-1, PD-L1, PD-L2, CTLA-4, LAG3, CD3, CD8, CD19, and IDO1, TMB, and MSI high status. In this analysis, 217 ICI-treated patients were primarily analyzed. Survival time was defined as the time from initiation of ICI therapy to death or last follow-up date (OS) or to disease progression, death, or last follow-up date (PFS); all patients were included and patients who had not progressed were censored on the last date of follow-up for PFS or the data cutoff date, whichever came first; patients who had not died were censored on the last date of follow-up for OS or the data cutoff date, whichever came first (data cutoff date, June 2022). For CXCL13 expression classification, the overall survival of 272 patients with advanced disease who were not treated with ICIs was also analyzed to evaluate the prognostic impact of CXCL13 RNA expression. In this analysis, OS was defined as the time from advanced disease diagnosis to death or last follow-up date. Survival differences between the two RNA expression groups of each immunoregulatory factor were assessed using the Kaplan-Meier method with a log-rank test. All statistical analyses were performed using R version 4.2.3 on the RStudio platform (version 2023.9.1.494).

## Supplementary information

Supplementary Information

## Data Availability

Any data to reanalyze the data reported in this paper is available from the corresponding authors upon request. The data supporting the findings of this study are available in the article and supplementary materials.

## Code availability

No custom codes were used for the analyses, and all analyses were performed using publicly available R packages. Only general codes were used for analyses, and thus, these were not deposited in a public repository.
