# Supplementary material for: Orchestration of B and T cells predicts prolonged survival after cancer immune checkpoint inhibitor therapy
Source: NPJ Precis Oncol. 2026 Apr 28;10:256. doi: 10.1038/s41698-026-01427-9 (PMC13328500; doi:10.1038/s41698-026-01427-9)
Supplement: Supplementary file 1 — Supplementary Information [file 41698_2026_1427_MOESM1_ESM.pdf]

Supplementary material

Orchestration of B and T cells predicts prolonged survival after cancer immune checkpoint inhibitor therapy

Authors: Yu Fujiwara, Shumei Kato, Daisuke Nishizaki, Hirotaka Miyashita, Suzanna Lee, Taylor J. Jensen, Paul DePietro, RJ Seager, Sadakatsu Ikeda, Razelle Kurzrock

Supplementary Table 1. The evaluated immunoregulatory markers.
Supplementary Table 2. Clinical characteristics of patients with longer (6 months or more) and shorter (less than 6 months) progression-free survival after immune checkpoint inhibitors.
Supplementary Table 3. Clinical characteristics of patients with longer (1 year or more) and shorter (less than 1 year) overall survival after immune checkpoint inhibitors.

Supplementary Figure 1. Heatmap of B and T cell transcriptome expression.
Supplementary Figure 2. Correlation matrix of B and T cell markers.
Supplementary References.
Reporting Checklist. STROBE checklist for cohort studies.

Supplementary Table 1. The evaluated immunoregulatory markers.

Category	Marker	Function in the tumor immune microenvironment	Supplementary References
Pan B cell	CD19	Expressed on all stages of B cells	[1]
	CD20	Expressed on naïve and memory B cells	[1]
	CD79a	Expressed on B-lineage cells including plasma cells	[1]
Memory B cell	CD80	A marker of memory B cells, it binds to CD28 and CTLA-4	[1]
	CD27	A memory B cell marker, works as a co-stimulatory checkpoint on T cells	[1]
Plasma cell	CD38	Plasma cell marker	[1]
	p63	Rough endoplasmic reticulum-associated protein expressed highly on plasma cells	[2]
TLS	IL-21	Enhance the differentiation of B cells into plasma cells, and the development of follicular helper cells, is involved in TLS formation	[3]
	CCL21	A chemokine that plays a role in the formation and maintenance of TLSs	[3]
	CXCL13	A cytokine that facilitates the organization and formation of TLSs	[3]
Regulatory B cell	CD40	A marker of regulatory B cells and works as a ligand of co-stimulatory immune checkpoints. It also has a role to induce TLSs	[3, 4]
	Granzyme B	B cells use granzyme B to regulate other cells including the suppression of adjacent effector T cells	[5]
	IL-10	IL-10 producing B cells suppress excessive immune responses and inhibits cytotoxic T cells	[5]
	TGF-β	Suppresses pro-inflammatory lymphocytes when produced by B cells	[5]
	TNF	Enhances the expansion of regulatory B cells	[5]
Pan T cell	CD3	Pan T cell marker	[6]
Helper T cell	CD4	Commonly used helper T cell marker	[6]
Cytotoxic T cell	CD8	Commonly used cytotoxic T cell marker	[6]
Regulatory T cell	FOXP3	Commonly used marker for regulatory T cell	[7]
Co-stimulatory immune checkpoint	4-1BB (CD137)	Expressed on activated T and NK cell, enhances their immunity	[8]
	CD28	Expressed on T cells and provide co-stimulatory signals for T cell activation	[8]
	CD40L (CD154)	Expressed on activated T cells, interacts with CD40 on B cells and antigen-presenting cells	[8]
	CD70	A ligand for CD27, promotes T cell survival and memory formation	[9]
	ICOS	Enhances T cell effector function	[8]
	OX40	Expressed on activated T cells, promoting T cell survival	[8]
Co-inhibitory immune checkpoint	BTLA	An inhibitory receptor expressed on T and B cells, suppresses T cell activation and B cell signaling	[8]

	CTLA-4	Expressed on activated and regulatory T cells, suppresses immune response by competing with CD28	[8]
	LAG3	Inhibits T cell proliferation and cytokine production, expressed on exhausted T cells	[8]
	PD-1	A key inhibitory immune checkpoint, expressed on exhausted T cells	[8]
	PD-L1	Ligand of PD-1 to suppress T cell function, upregulated on tumor and antigen-presenting cells	[8]
	PD-L2	Ligand of PD-1, expressed on dendritic cells, macrophages, and tumor cells	[8]
	TIGIT	An inhibitory receptor on T cells, inducing suppression of T cell activation	[8]
	TIM3	Expressed on exhausted T cells, impairs effector T cell function	[8]
	VISTA	An inhibitory checkpoint expressed on myeloid and regulatory T cells	[8]
Immune metabolite	IDO1	Catabolize tryptophan into its metabolites, upregulated in an inflamed environment to suppress effector T cells	[10, 11]

Abbreviations: TLS, tertiary lymphoid structure.

References are summarized in Supplementary References.

Supplementary Table 2. Clinical characteristics of patients with longer (6 months or more) and shorter (less than 6 months) progression-free survival after immune checkpoint inhibitors.

**Factor**	**Group**	**Longer PFS (N=82)**	**Shorter PFS (N=126)**	**P value**
Age, years (median [IQR])		64.98 [54.05, 71.26]	61.00 [47.83, 68.77]	**0.020**
Sex (%)	Female	48 (58.5%)	69 (54.8%)	0.668
	Male	34 (41.5%)	57 (45.2%)	
Cancer status at mRNA expression analysis (%)	Metastatic	38 (46.3%)	68 (54.0%)	0.510
	Primary	42 (51.2%)	54 (42.9%)	
	Recurrent	2 (2.4%)	4 (3.2%)	
ICI combined with other systemic therapy (%)	Yes	47 (57.3%)	80 (63.5%)	0.386
	No	35 (42.7%)	46 (36.5%)	
ICI type (%)	CTLA-4	1 (1.2%)	1 (0.8%)	0.419
	Dual ICIs (PD-1+CTLA-4)	8 (9.8%)	8 (6.3%)	
	PD-1	64 (78.0%)	109 (86.6%)	
	PD-L1	9 (11.0%)	8 (6.3%)	
Cancer type (%)	Basal Cell Carcinoma	1 (1.2%)	0 (0.0%)	N/A
	Bladder Cancer	0 (0.0%)	3 (2.4%)	
	Breast Cancer	6 (7.3%)	12 (9.5%)	
	Cervical Cancer	2 (2.4%)	1 (0.8%)	
	Colorectal Cancer	18 (22.0%)	32 (25.4%)	
	Esophageal Cancer	3 (3.7%)	7 (5.6%)	
	Gallbladder and Extrahepatic Bile Duct Cancers	2 (2.4%)	0 (0.0%)	
	Head and Neck Cancer	2 (2.4%)	3 (2.4%)	
	Gastric Cancer	4 (4.9%)	6 (4.8%)	
	Liver and Bile Duct Cancer	3 (3.7%)	6 (4.8%)	
	Lung Cancer	6 (7.3%)	7 (5.6%)	
	Melanoma	3 (3.7%)	3 (2.4%)	
	Mesothelioma	0 (0.0%)	2 (1.6%)	
	Neuroendocrine Tumors	3 (3.7%)	2 (1.6%)	
	Ocular melanoma	1 (1.2%)	0 (0.0%)	
	Ovarian Cancer	6 (7.3%)	12 (9.5%)	
	Pancreatic Cancer	6 (7.3%)	9 (7.1%)	
	Prostate Cancer	1 (1.2%)	1 (0.8%)	
	Renal cell carcinoma	1 (1.2%)	1 (0.8%)	
	Sarcoma	6 (7.3%)	3 (2.4%)	
	Small Intestine Cancer	0 (0.0%)	1 (0.8%)	

	Squamous Cell Carcinoma of the Skin	0 (0.0%)	3 (2.4%)	
	Thyroid Cancer	0 (0.0%)	3 (2.4%)	
	Unknown Primary Cancer	2 (2.4%)	3 (2.4%)	
	Uterine Cancer	6 (7.3%)	6 (4.8%)	
TMB (median [IQR]) (mutation/mb)		5.65 [3.40, 7.88]	4.40 [2.60, 6.90]	0.104
MSI status (%)	MSS	67 (81.7%)	102 (81.0%)	**0.024**<br>**MSI-H was associate with longer PFS**
	MSI-H	7 (8.5%)	2 (1.6%)	
	N/A	8 (9.8%)	22 (17.5%)	

Footnote: Any patients censored before 6 months were excluded from the analysis. We did not perform a statistical analysis to compare the rate of cancer types between two groups as each cancer type consists of a small number of patients in two groups.

Abbreviations: CTLA-4, cytotoxic T-lymphocyte associated protein 4; ICI, immune checkpoint inhibitor; IQR, interquartile range; MSI, microsatellite instability; MSS microsatellite stable; N/A, not available/applicable; PD-1, programmed cell death protein 1; PD-L1, programmed death-ligand 1; PFS, progression-free survival; TMB, tumor mutational burden.

Supplementary Table 3. Clinical characteristics of patients with longer (1 year or more) and shorter (less than 1 year) overall survival after immune checkpoint inhibitors.

Factor	Group	Longer OS (N=100)	Shorter OS (N=80)	P value
Age, years (median [IQR])		61.66 [51.63, 70.90]	62.58 [50.39, 68.96]	0.889
Sex (%)	Female	60 (60.0%)	43 (53.8%)	0.45
	Male	40 (40.0%)	37 (46.2%)	
Cancer status at mRNA expression analysis (%)	Metastatic	52 (52.0%)	39 (48.8%)	0.747
	Primary	46 (46.0%)	38 (47.5%)	
	Recurrent	2 (2.0%)	3 (3.8%)	
ICI combined with other systemic therapy (%)	Yes	55 (55.0%)	54 (67.5%)	0.094
	No	45 (45.0%)	26 (32.5%)	
ICI type (%)	CTLA-4	2 (2.0%)	0 (0.0%)	0.065
	Dual ICIs (PD-1+CTLA-4)	11 (11.0%)	4 (5.0%)	
	PD-1	77 (77.0%)	73 (91.2%)	
	PD-L1	10 (10.0%)	3 (3.8%)	
Cancer type (%)	Basal Cell Carcinoma of the Skin	1 (1.0%)	0 (0.0%)	N/A
	Bladder Cancer	1 (1.0%)	0 (0.0%)	
	Breast Cancer	11 (11.0%)	5 (6.2%)	
	Cervical Cancer	2 (2.0%)	0 (0.0%)	
	Colorectal Cancer	21 (21.0%)	25 (31.2%)	
	Esophageal Cancer	1 (1.0%)	5 (6.2%)	
	Gallbladder and Extrahepatic Bile Duct Cancers	1 (1.0%)	1 (1.2%)	
	Head and Neck Cancer	4 (4.0%)	1 (1.2%)	
	Kidney and Renal Pelvis Cancer	2 (2.0%)	0 (0.0%)	
	Liver and Bile Duct Cancer	3 (3.0%)	6 (7.5%)	
	Lung Cancer	8 (8.0%)	3 (3.8%)	
	Melanoma	6 (6.0%)	0 (0.0%)	
	Mesothelioma	0 (0.0%)	2 (2.5%)	
	Neuroendocrine Tumors	3 (3.0%)	1 (1.2%)	
	ocular melanoma	1 (1.0%)	0 (0.0%)	
	Ovarian Cancer	10 (10.0%)	6 (7.5%)	
	Pancreatic Cancer	5 (5.0%)	8 (10.0%)	
	Prostate Cancer	1 (1.0%)	1 (1.2%)	
	Sarcoma	6 (6.0%)	2 (2.5%)	
	Small Intestine Cancer	0 (0.0%)	1 (1.2%)	
	Squamous Cell Carcinoma of the Skin	0 (0.0%)	2 (2.5%)	

	Stomach Cancer	3 (3.0%)	6 (7.5%)	
	Thyroid Cancer	1 (1.0%)	0 (0.0%)	
	Unknown Primary Cancer	2 (2.0%)	2 (2.5%)	
	Uterine Cancer	7 (7.0%)	3 (3.8%)	
TMB (median [IQR]) (mutation/mb)		5.20 [3.40, 6.97]	5.20 [3.40, 7.20]	0.961
MSI status (%)	MSS	83 (83.0%)	66 (82.5%)	**0.008 MSI-H was associated with longer OS**
	MSI-H	8 (8.0%)	0 (0.0%)	
	N/A	9 (9.0%)	14 (17.5%)	

Footnote: Any patients censored before 1 year were excluded from the analysis. We did not perform a statistical analysis to compare the rate of cancer types between two groups as each cancer type consists of a small number of patients in two groups.

Abbreviations: CTLA-4, cytotoxic T-lymphocyte associated protein 4; ICI, immune checkpoint inhibitor; IQR, interquartile range; MSI, microsatellite instability; MSS microsatellite stable; N/A, not available/applicable; OS, overall survival; PD-1, programmed cell death protein 1; PD-L1, programmed death-ligand 1; TMB, tumor mutational burden.

Supplementary Figure 1. Heatmap of B and T cell transcriptome expression
(a) Patients with longer progression-free survival (6 months or more, **N=82**). (b) Patients with shorter progression-free survival (less than 6 months, **N=126**). (c) Patients with longer overall survival (1 year or more, **N=100**). (d) Patients with shorter overall survival (less than 1 year, **N=80**). (e) All 514 patients in the study. Any patients censored before 6 months in (a) and (b), and any patients censored before 1 year in (c) and (d), were excluded from the analysis. The color key shows the RNA percentile rank of expression. Figures suggest that patients with PFS of 6 months or more and those with OS of 1 year or more are more likely to express high levels of B and T cell markers and certain checkpoints, as reflected by the red cluster.

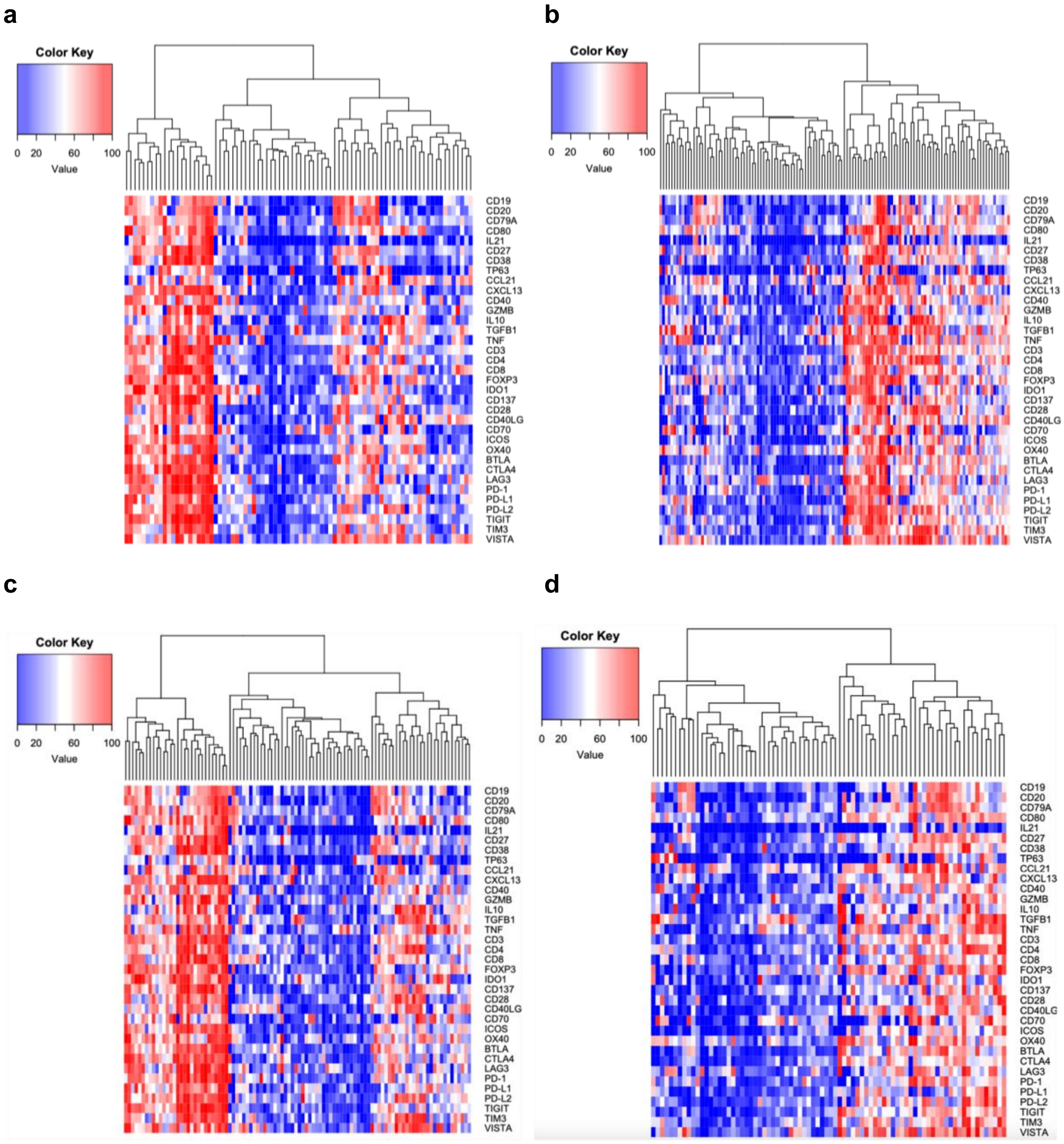

e

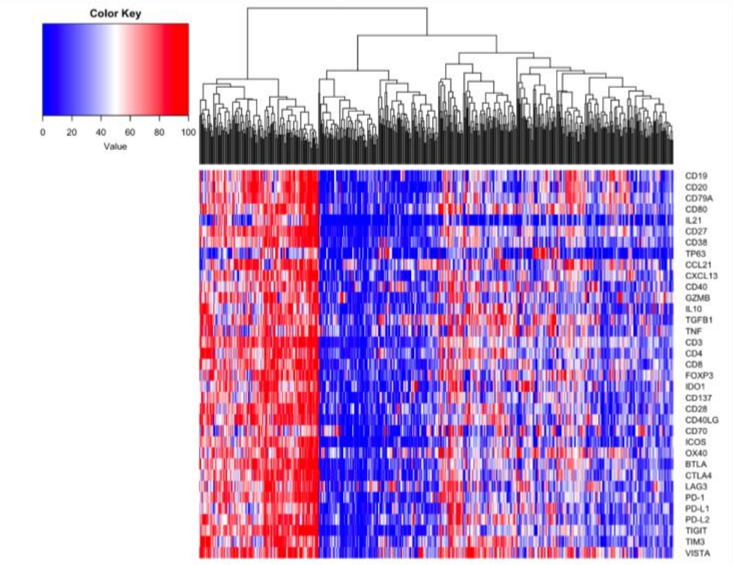

Color Key
0
20
40
60
80
100
Value
CD19
CD20
CD79A
CD80
IL21
CD27
CD38
TP63
CCL21
CXCL13
CD40
GZMB
IL10
TGFB1
TNF
CD3
CD4
CD8
FOXP3
IDO1
CD137
CD28
CD40LG
CD70
ICOS
OX40
BTLA
CTLA4
LAG3
PD-1
PD-L1
PD-L2
TIGIT
TIM3
VISTA

Supplementary Figure 2. Correlation matrix of B and T cell markers

(a) Correlation matrix of B and T cell markers in patients with progression-free survival of 6 months or more after immune checkpoint inhibitor therapy (**N=82).** Spearman correlations (bar on the right) were calculated to assess the co-expression of selected 35 immunoregulatory factors (see **Supplementary Table 1**) including B and T cell markers and co-stimulatory and inhibitory immune checkpoints. Red color means stronger correlation and blue color means weaker correlation. Hierarchical clustering method was used to visualize the landscape of correlations among these factors. Strong correlation among checkpoints (CTLA4, ICOS, BTLA, TIGIT, PD-1), tertiary lymphoid structures (CXCL13, IL-21), and T cells (CD3, CD8) was observed. Any patients censored before 6 months were excluded from the analysis.

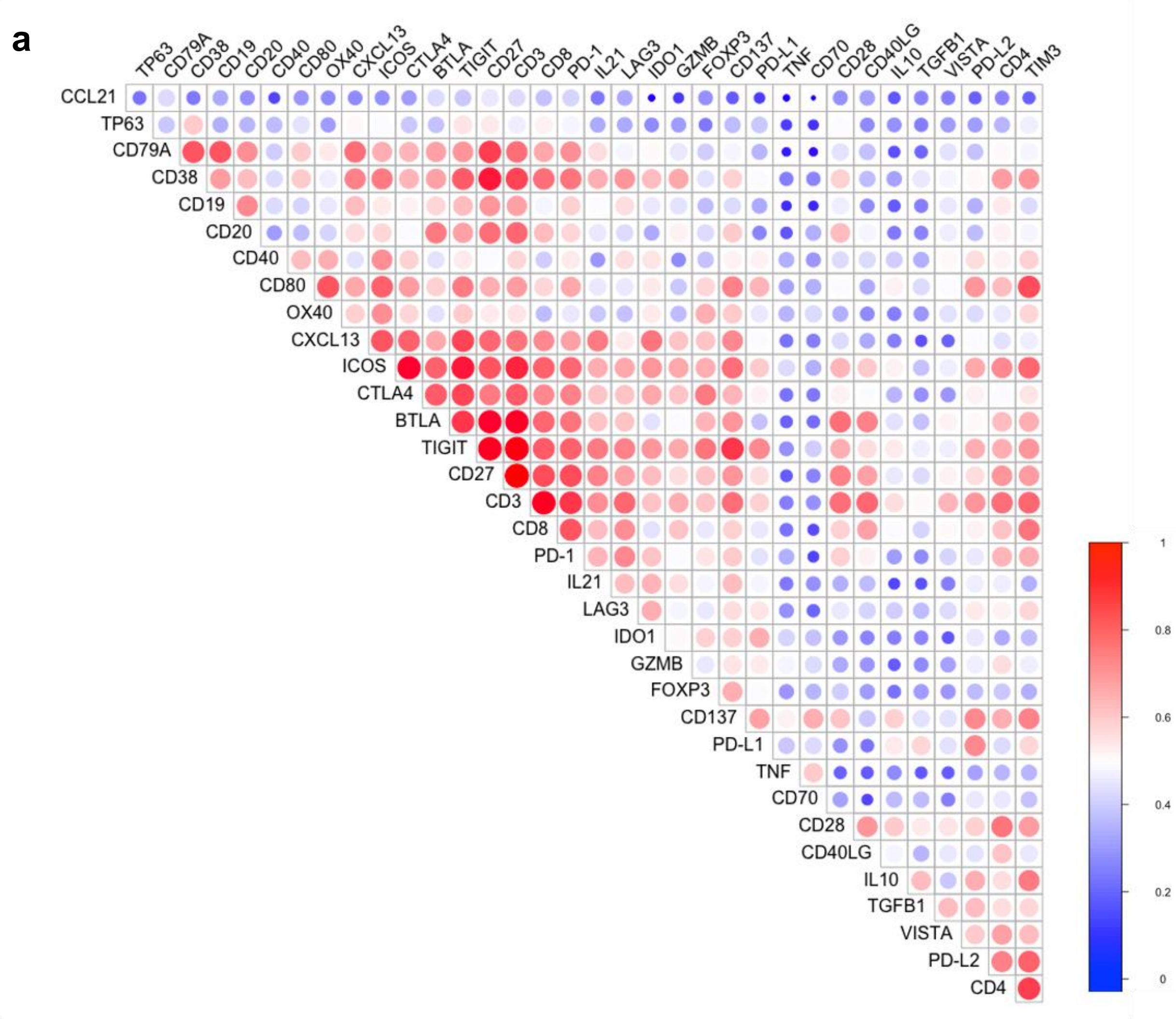

(b) Correlation matrix of B and T cell markers in patients with progression-free survival of less than 6 months (**N=126**).

Spearman correlations (bar on the left) were calculated to assess the co-expression of selected 35 immunoregulatory factors (see **Supplementary Table 1**) including B and T cell markers and co-stimulatory and inhibitory immune checkpoints. Red color means stronger correlation and blue color means weaker correlation. Hierarchical clustering method was used to visualize the landscape of correlations among these factors. Strong correlations among checkpoints (BTLA, CTLA-4, ICOS, CD40LG, TIGIT, CD28) were observed but not among tertiary lymphoid structures (CXCL13, IL-21) and T cells (CD3, CD8) in patients with PFS of less than 6 months. Any patients censored before 6 months were excluded from the analysis.

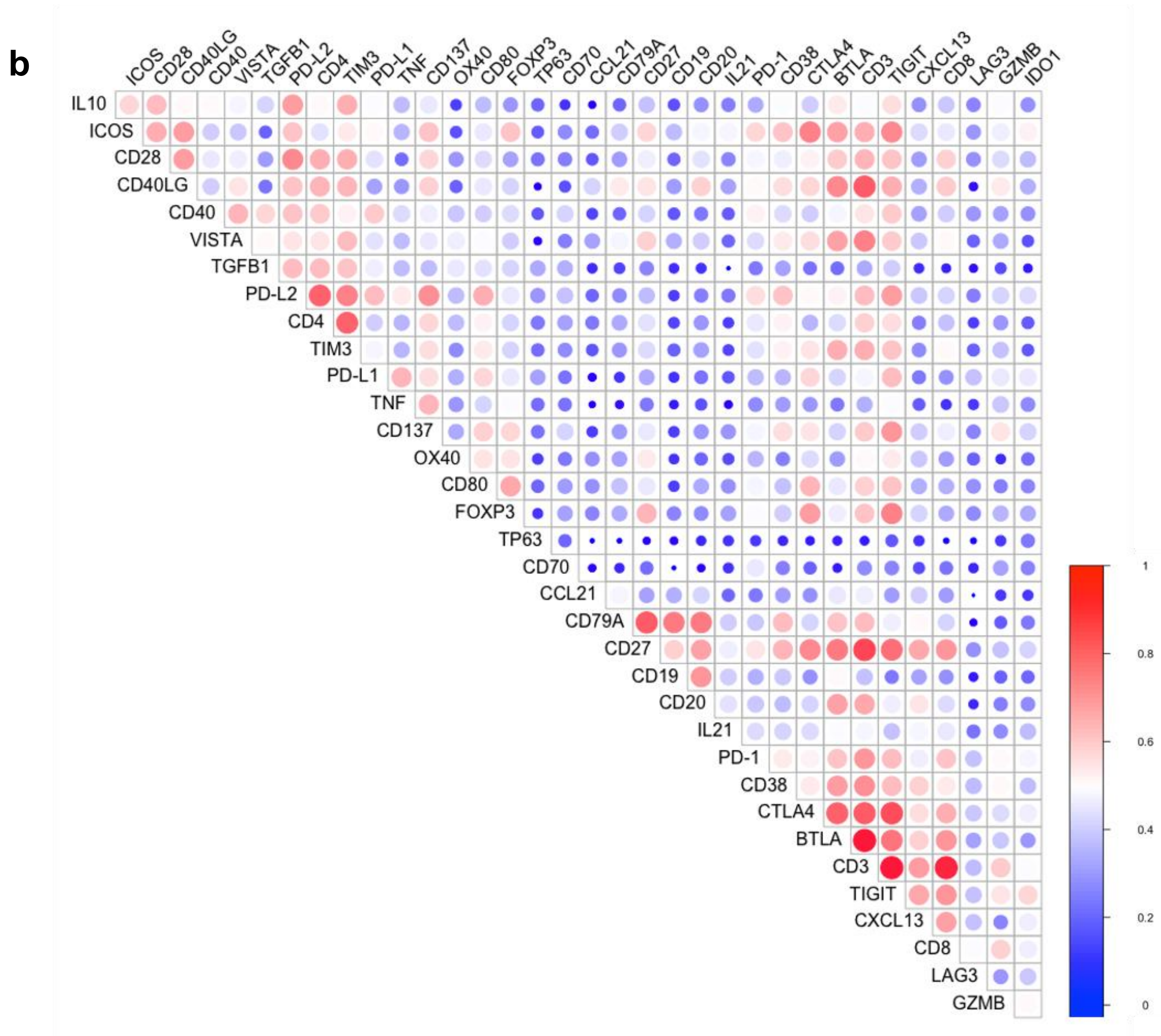

(c) Correlation matrix of B and T cell markers in patients with overall survival of 1 year or more after immune checkpoint inhibitor therapy (**N=100).**

Spearman correlations (bar on the right) were calculated to assess the co-expression of selected 35 immunoregulatory factors (see **Supplementary Table 1**) including B and Tcell markers and co-stimulatory and inhibitory immune checkpoints. Red color means stronger correlation and blue color means weaker correlation. Hierarchical clustering method was used to visualize the landscape of correlations among these factors. Strong correlation among checkpoints (CTLA4, ICOS, BTLA, TIGIT, PD-1), tertiary lymphoid structures (CXCL13, IL-21), and T cells (CD3, CD8) was observed. Any patients censored before 1 year were excluded from the analysis.

c

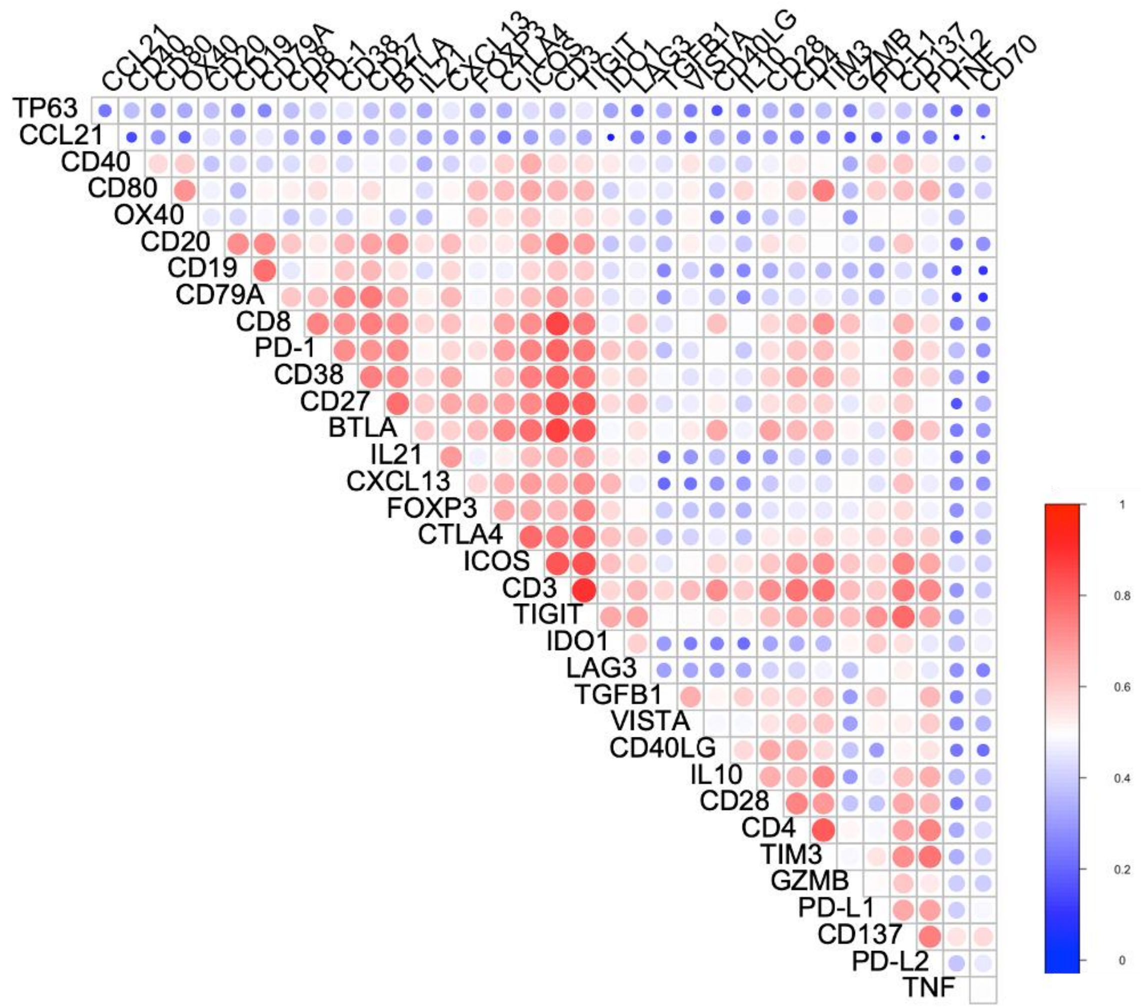

(d) Correlation matrix of B and T cell markers in patients with overall survival of less than 1 year after immune checkpoint inhibitor therapy (**N=80).**

Spearman correlations (bar on the left) were calculated to assess the co-expression of selected 35 immunoregulatory factors (see **Supplementary Table 1**) including B and T cell markers and co-stimulatory and inhibitory immune checkpoints. Red color means stronger correlation and blue color means weaker correlation. Hierarchical clustering method was used to visualize the landscape of correlations among these factors. Strong correlations among checkpoints (BTLA, CTLA-4, TIGIT) and T cells (CD3, CD8) were observed but not among tertiary lymphoid structures (CXCL13, IL-21) in patients with overall survival of less than one year. Any patients censored before one year were excluded from the analysis.

d

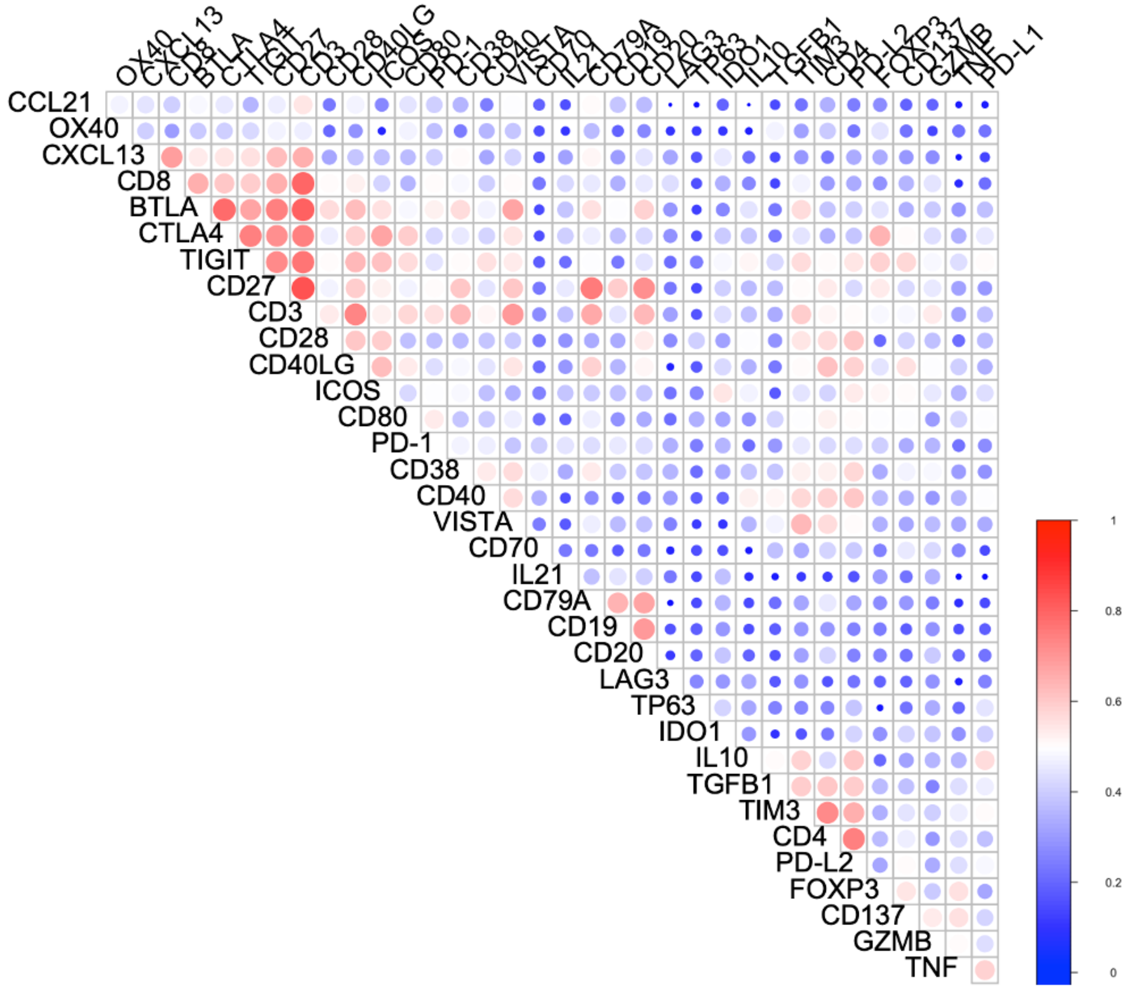

(e) Correlation matrix of B and T cell markers in 514 patients enrolled in this study.

Spearman correlations (bar on the left) were calculated to assess the co-expression of selected 35 immunoregulatory factors (see **Supplementary Table 1**) including B and T cell markers and co-stimulatory and inhibitory immune checkpoints. Red color means stronger correlation and blue color means weaker correlation. Hierarchical clustering, using Ward 2 method, was used to visualize the landscape of correlations among these factors. This figure shows strong correlations among certain inhibitory checkpoints (CTLA-4, PD-1, BTLA, and TIGIT), T cell markers (CD3 and CD8), and CXCL13, as represented in a red color at the left top side of the heatmap. This suggests that these factors are dynamically interactive together in patients with solid tumors regardless of the degree of response to immune checkpoint inhibitors.

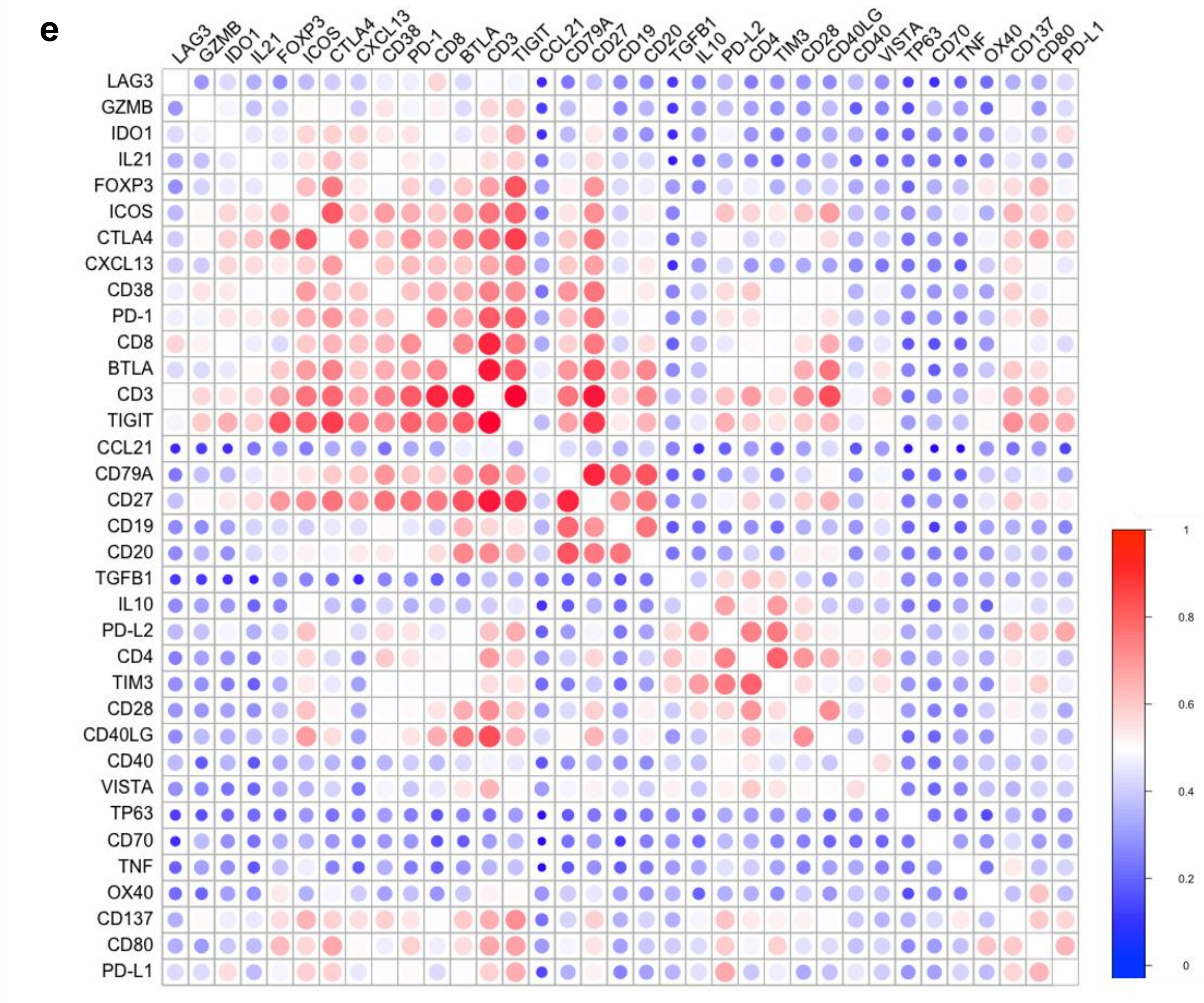

Supplementary References.

1. Maecker HT, McCoy JP, Nussenblatt R. Standardizing immunophenotyping for the Human Immunology Project. Nat Rev Immunol. 2012;12(3):191-200.
2. Banham AH, Turley H, Pulford K, Gatter K, Mason DY. The plasma cell associated antigen detectable by antibody VS38 is the p63 rough endoplasmic reticulum protein. J Clin Pathol. 1997;50(6):485-9.
3. Fridman WH, Meylan M, Pupier G, Calvez A, Hernandez I, Sautès-Fridman C. Tertiary lymphoid structures and B cells: An intratumoral immunity cycle. Immunity. 2023;56(10):2254-69.
4. Fridman WH, Zitvogel L, Sautès-Fridman C, Kroemer G. The immune contexture in cancer prognosis and treatment. Nat Rev Clin Oncol. 2017;14(12):717-34.
5. Downs-Canner SM, Meier J, Vincent BG, Serody JS. B Cell Function in the Tumor Microenvironment. Annu Rev Immunol. 2022;40:169-93.
6. Weinberg RA. The Biology of Cancer (3rd edition): WW Norton & Company; 2023.
7. Rudensky AY. Regulatory T cells and Foxp3. Immunol Rev. 2011;241(1):260-8.
8. Sharma P, Goswami S, Raychaudhuri D, Siddiqui BA, Singh P, Nagarajan A, et al. Immune checkpoint therapy-current perspectives and future directions. Cell. 2023;186(8):1652-69.
9. Jacobs J, Deschoolmeester V, Zwaenepoel K, Rolfo C, Silence K, Rottey S, et al. CD70: An emerging target in cancer immunotherapy. Pharmacol Ther. 2015;155:1-10.
10. Fujiwara Y, Kato S, Nesline MK, Conroy JM, DePietro P, Pabla S, et al. Indoleamine 2,3-dioxygenase (IDO) inhibitors and cancer immunotherapy. Cancer Treat Rev. 2022;110:102461.
11. Spranger S, Spaapen RM, Zha Y, Williams J, Meng Y, Ha TT, et al. Up-regulation of PD-L1, IDO, and T(regs) in the melanoma tumor microenvironment is driven by CD8(+) T cells. Sci Transl Med. 2013;5(200):200ra116.

Reporting Checklist. STROBE checklist for cohort studies.

	Item No	Recommendation	Page No
**Title and abstract**	1	(*a*) Indicate the study's design with a commonly used term in the title or the abstract	2
		(*b*) Provide in the abstract an informative and balanced summary of what was done and what was found	2
**Introduction**			
Background/rationale	2	Explain the scientific background and rationale for the investigation being reported	3, 4
Objectives	3	State specific objectives, including any prespecified hypotheses	4
**Methods**			
Study design	4	Present key elements of study design early in the paper	10, 11
Setting	5	Describe the setting, locations, and relevant dates, including periods of recruitment, exposure, follow-up, and data collection	10
Participants	6	(*a*) Give the eligibility criteria, and the sources and methods of selection of participants. Describe methods of follow-up	10
		(*b*) For matched studies, give matching criteria and number of exposed and unexposed	NA
Variables	7	Clearly define all outcomes, exposures, predictors, potential confounders, and effect modifiers. Give diagnostic criteria, if applicable	11, 12
Data sources/ measurement	8*	For each variable of interest, give sources of data and details of methods of assessment (measurement). Describe comparability of assessment methods if there is more than one group	11, 12
Bias	9	Describe any efforts to address potential sources of bias	12
Study size	10	Explain how the study size was arrived at	10
Quantitative variables	11	Explain how quantitative variables were handled in the analyses. If applicable, describe which groupings were chosen and why	11, 12
Statistical methods	12	(*a*) Describe all statistical methods, including those used to control for confounding	11, 12
		(*b*) Describe any methods used to examine subgroups and interactions	12
		(*c*) Explain how missing data were addressed	20, 22
		(*d*) If applicable, explain how loss to follow-up was addressed	NA
		(*e*) Describe any sensitivity analyses	NA
**Results**			
Participants	13*	(a) Report numbers of individuals at each stage of study—eg numbers potentially eligible, examined for eligibility, confirmed eligible, included in the study, completing follow-up, and analysed	4,5
		(b) Give reasons for non-participation at each stage	NA
		(c) Consider use of a flow diagram	NA
Descriptive data	14*	(a) Give characteristics of study participants (eg demographic, clinical, social) and information on exposures and potential confounders	4,5
		(b) Indicate number of participants with missing data for each variable of interest	20, 22
		(c) Summarise follow-up time (eg, average and total amount)	Fig 4
Outcome data	15*	Report numbers of outcome events or summary measures over time	6, 7

Main results	16	(*a*) Give unadjusted estimates and, if applicable, confounder-adjusted estimates and their precision (eg, 95% confidence interval). Make clear which confounders were adjusted for and why they were included	6,7
		(*b*) Report category boundaries when continuous variables were categorized	5, 6, 7
		(*c*) If relevant, consider translating estimates of relative risk into absolute risk for a meaningful time period	NA
Other analyses	17	Report other analyses done—eg analyses of subgroups and interactions, and sensitivity analyses	5, 6
**Discussion**			
Key results	18	Summarise key results with reference to study objectives	7
Limitations	19	Discuss limitations of the study, taking into account sources of potential bias or imprecision. Discuss both direction and magnitude of any potential bias	8, 9
Interpretation	20	Give a cautious overall interpretation of results considering objectives, limitations, multiplicity of analyses, results from similar studies, and other relevant evidence	7-10
Generalisability	21	Discuss the generalisability (external validity) of the study results	9, 10
**Other information**			
Funding	22	Give the source of funding and the role of the funders for the present study and, if applicable, for the original study on which the present article is based	13

*Give information separately for exposed and unexposed groups.

Note: An Explanation and Elaboration article discusses each checklist item and gives methodological background and published examples of transparent reporting. The STROBE checklist is best used in conjunction with this article (freely available on the Web sites of PLoS Medicine at http://www.plosmedicine.org/, Annals of Internal Medicine at http://www.annals.org/, and Epidemiology at http://www.epidem.com/). Information on the STROBE Initiative is available at http://www.strobe-statement.org.
